# Compound Fault Diagnosis of Rolling Bearing Based on Singular Negentropy Difference Spectrum and Integrated Fast Spectral Correlation

**DOI:** 10.3390/e22030367

**Published:** 2020-03-23

**Authors:** Guiji Tang, Tian Tian

**Affiliations:** Department of Mechanical Engineering, North China Electric Power University, Baoding 071000, China; tanggjlk@ncepu.edu.cn

**Keywords:** singular value decomposition, singular negentropy difference spectrum, integrated fast spectral correlation, rolling bearing, composite fault, fault separation

## Abstract

Compound fault diagnosis is challenging due to the complexity, diversity and non-stationary characteristics of mechanical complex faults. In this paper, a novel compound fault separation method based on singular negentropy difference spectrum (SNDS) and integrated fast spectral correlation (IFSC) is proposed. Firstly, the original signal was de-noised by SNDS which improved the noise reduction effect of singular difference spectrum by introducing negative entropy. Secondly, the de-noised signal was analyzed by fast spectral correlation. Finally, IFSC took the fourth-order energy as the index to determine the resonance band and separate the fault features of different single fault. The proposed method is applied to analyze the simulated compound signals and the experimental vibration signals, the results show that the proposed method has excellent performance in the separation of rolling bearing composite faults.

## 1. Introduction

**Figure 1:**
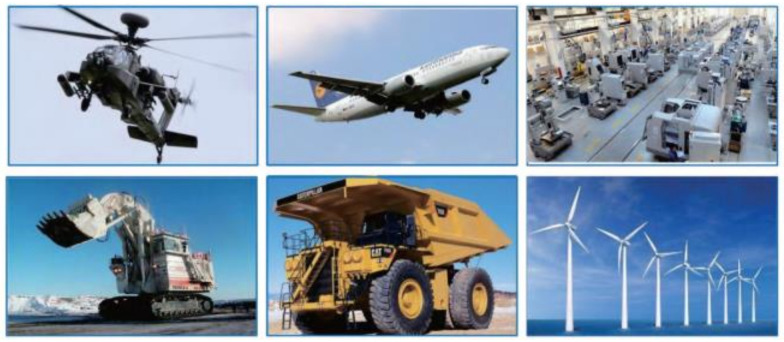
Some application examples of rotating machinery [1].

Rotating machinery is widely used in modern industries, such as helicopters, airplanes, machining centers, track loaders, mining tracks and wind turbines [1], as shown in Figure 1. Rolling bearing is one of the most common components in rotating machines. Whether the rolling bearing runs normally is directly related to the running state of the whole rotating machinery [2,3]. Due to the bad working environment and long-term working conditions, several key parts of the rolling bearing are easy to be damaged at the same time, resulting in composite faults [4]. Composite faults are more harmful to machinery than the single faults, so it is important to diagnose early complex faults. However, all kinds of fault features are closely coupled and interfere with each other, which makes the separation of rolling bearing composite faults more challenging [5].

A variety of technical system of rotating machinery fault diagnosis has been formed, such as vibration analysis [6], acoustic emission analysis [7,8], electrical signal analysis [9] and oil analysis [10]. Among them, fault diagnosis based on vibration analysis is the most widely used. The separation of rolling bearing composite faults based on vibration analysis is a hot issue. At present, researchers have proposed some methods to extract the composite fault features of rolling bearings, including resonance demodulation, spectral kurtosis, wavelet analysis, maximum correlated kurtosis deconvolution and variational mode decomposition. For example, Wang et al. [11] solved the compound fault problem by combining the meshing resonance and spectral kurtosis. Wang et al. [12] applied the adaptive spectral kurtosis to multi-fault detection. Dhamande et al. [13] proposed a fault diagnosis method combining continuous and discrete wavelet transform. He et al. [14] proposed an adaptive redundant multiwavelet packet method to diagnose compound fault. Teng et al. [15] established a novel vibration model and used empirical wavelet transform to find multiple fault feature. Lyu et al. [16] proposed an improved maximum correlated kurtosis deconvolution method based on quantum genetic algorithm to diagnose compound fault. Miao et al. [17] developed an improved parameter-adaptive variational mode decomposition for identification of compound fault. Pan et al. [18] utilized symplectic geometry mode decomposition to decompose complex signal and got a good result.

Singular value decomposition (SVD) is a nonlinear filtering method widely used in signal denoising and fault diagnosis [19,20,21]. Conventional noise reduction methods based on SVD require the feature signal to be the main component of the signal. In this way, the prominent singular values obtained by singular value decomposition correspond to the signal space, while the smaller singular values correspond to the noise space. As long as the dimensionality reduction matrix corresponding to the more prominent singular values of the first few orders is retained, the signal after noise reduction can be obtained. However, when the background noise is very strong, the characteristic signal is completely submerged by the noise, and the singular value cannot be obtained after singular value decomposition, the number of useful component signals cannot be determined by singular value difference spectrum method. In order to overcome this problem, singular negentropy difference spectrum (SNDS) was proposed in this paper. 

Cyclostationarity is a widespread physical phenomenon in rotating machinery, which is manifested by the correlation between the spectrum lines of vibration signals [22]. The vibration signal of rolling bearing is a typical cycle stationary signal and its characteristic cyclic frequency with harmonic frequency components. According to the different statistical characteristic parameters, cyclostationary stochastic processes can be divided into first, second and higher order cyclostationary processes. Spectral correlation (SC) is the main tool of second-order cyclostationary analysis. SC shows the whole structure of modulation and carrier in the signal in the form of bispectrum [23]. Fast spectral correlation (FSC) is a compromised algorithm between performance and computational efficiency which is an improved version of spectral correlation based on short time Fourier transform [24]. FSC can effectively identify the periodic impact components in the bearing, and has a high calculation efficiency. Therefore, FSC is chosen as the fault feature extraction method in this paper. However, FSC can only extract the composite fault features together, it can not separate the composite fault features. Based on FSC, integrated fast spectral correlation (IFSC) is proposed. It can identify the resonance band through the fourth order energy, and separate the composite fault. In the research of bearing composite fault diagnosis, it is found that the existence of noise affects the separation of composite fault. Therefore, in this paper, SNDS was used to de-noise the composite fault signal, and then IFSC separated the de-noised signal.

The rest of the paper is organized as follows. Section 2 describes the principles of SVD, SNDS and IFSC. Section 3 shows the specific process of the proposed method. In Section 4 and Section 5, the effectiveness of the proposed method is verified by simulation signal and experimental signal, respectively. The conclusions are presented in Section 6.

## 2. Basic Theory 

### 2.1. Singular Negentropy Difference Spectrum

#### 2.1.1. SVD

Assuming that the original discrete signal *X* = [*x*(1), *x*(2), …, *x*(*N*)], based on the theory of phase space reconstruction, the Hankel matrix **A** is constructed as follows [19]:

(1) $$A=\left[\begin{array}{cccc} x(1) & x(2) & \cdots & x(n)\\ x(2) & x(3) & \cdots & x(n+1)\\ \vdots & \vdots & \vdots & \vdots \\ x(N-n+1) & x(N-n+2) & \cdots & x(N) \end{array}\right],$$

where *N* is the length of the signal *X*, 1 < *n* < *N*. Let *m* = *N*-*n*+1, **A**∈**R***^m^*^×*n*^ which is the reconstructed attractor orbital matrix, and then perform singular value decomposition on it.

Perform singular value decomposition (SVD) on the matrix **A**, then the following equation can be obtained:

(2) $$A=USV^{T}=\sum_{i=1}^{k}u_{i}\lambda_{i}v_{i}^{T}=\sum_{i=1}^{k}A_{i},$$

where **U** = [*u*_1_, *u*_2_, …, *u_m_*]∈**R***^m^*^×*m*^, **V** = [*v*_1_, *v*_2_, …, *v_n_*]∈**R***^n^*^×*n*^ and *i* = 1, 2, …, *k*, *k* = min(*m*, *n*). **S** = diag(*λ*_1_, *λ*_2_,..., *λ_k_*) is a diagonal matrix arranged in descending order, and its diagonal element is the singular value of matrix **A**. A*_i_* is the submatrix corresponding to singular value *λ_i_* obtained via SVD decomposition.

The submatrix A*_i_* is inversely transformed to obtain the component signal *P_i_*. The result of linear superposition of *k* component signals obtained in this way is the original discrete signal *X*, that is:

(3) $$X=P_{1}+P_{2}+\ldots +P_{k},$$

#### 2.1.2. Singular Negentropy Difference Spectrum

Shannon Entropy is an effective index to quantitatively evaluate the uncertainties of signal or system state [25]. When the impact component appears, the Shannon entropy becomes smaller. The negentropy was defined, which is the negative value of the Shannon entropy, in order to keep the two changing regularities.

The definition of negentropy is as follows:

(4) $$E_{y}=\sum_{i=1}^{n}\left(p_{i}\log_{2}p_{i}\right),$$

where *y* = (*y*_1_, *y*_2_, …, *y_n_*) denotes a random variable, *p_i_* is the probability of *y_i_*.

In order to effectively preserve useful fault features and minimize the impact of noise, the singular negentropy difference spectrum (SNDS) based on SVD was introduced in this paper. The definition of SNDS is as follows:

(5) $$SNDS_{i}=E_{y}(i)-E_{y}(i+1),$$

where *E_y_*(*i*) means the negentropy of the first *i*-order reconstructed signal, *i* denotes the number of singular values.

**Figure 2:**
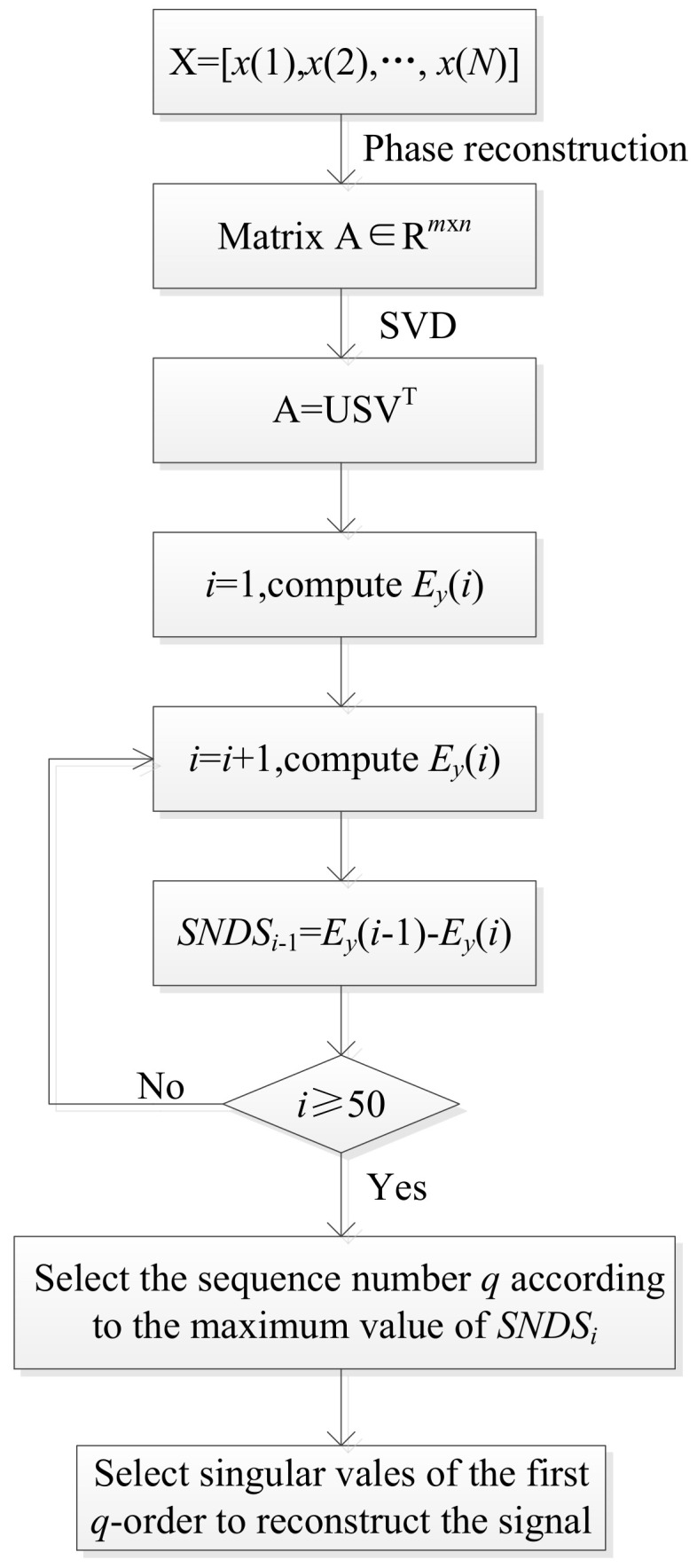
The schematic diagram of singular negentropy difference spectrum (SNDS).

The schematic diagram of SNDS is shown in Figure 2. When the negentropy of two reconstructed signals are quite different, a significant peak will appear in the difference spectrum. There will inevitably be a maximum peak in the whole difference spectrum, that is, the position *SNDS_q_* of the maximum mutation of the peak degree. The maximum break point *SNDS_q_* not only shows that there are abundant fault impact characteristics in the reconstructed signal, but also shows the boundary between the useful signal and the noise component. Thus, the first *q*-order reconstructed signal is selected.

### 2.2. Integrated Fast Spectral Correlation

#### 2.2.1. Fast Spectral Correlation

For a non-stationary random signal *x*(*t_n_*), its spectral correlation is given by [24]:

(6) $$SC_{x}(\alpha ,f)=\frac{1}{F_{s}{}^{2}}\sum_{n=-\infty }^{\infty }\sum_{\tau =-\infty }^{\infty }R_{x}(t_{n},\tau )e^{-j2\pi \alpha n\frac{1}{F_{s}}}e^{-j2\pi f\tau \frac{1}{F_{s}}},$$

where *F_s_* denotes the sampling frequency, *t_n_* means the sampling time, *t_n_* = *n* /*F_s_*, $R_{x}(t_{n},\tau )$ denotes the cyclic autocorrelation function of *x*(*t_n_*), τ denotes time delay, *α* means the cyclic frequency, *f* means frequency.

Fast spectral correlation (FSC) is an improved version of spectral correlation, which shorten the calculation time and improve the efficiency by short-time Fourier transform (STFT) [24]. STFT of signal *x*(*t_n_*) is as follows:

(7) $$X_{\mathrm{STFT}}(i,f_{k})=\sum_{n=0}^{N_{w}-1}x[iR+n]w[n]e^{-j2\pi n\frac{f_{k}}{F_{s}}},$$

The phase-corrected STFT might be presented as follows:

(8) $$X_{w}(i,f_{k})=\sum_{n=0}^{L-1}x[n]w[n-iR]e^{-j2\pi n\frac{f_{k}}{F_{s}}}=X_{\mathrm{STFT}}(i,f_{k})e^{-j2\pi iR\frac{f_{k}}{F_{s}}}$$

where *N_w_* is the window length of STFT, *R* denotes the block shift, *w*[*n*] means the function of time index *n*, *f_k_* denotes the *k*-th discrete frequency and *f_k_* = *k*Δ*f* (Δ*f* is the frequency resolution and it is expressed as Δ*f* = *F_s_*/*N_w_*), *L* is the length of signal *x*(*t_n_*).

Assume *f* = *f_k_* = *k*Δ*f* and *α* = *p*Δ*f* + *δ*, then *f*-*α* = *f_k_*-*α* ≈ *f_k_*_-*p*_, hence *α* ≈ *p*Δ*f*. Substitute these results into Equation (8)

(9) $$X_{w}(i,f_{k}-\alpha )\approx X_{w}(i,f_{k}{}_{-p})e^{j2\pi (\frac{\alpha }{F_{s}}-p\frac{\Delta f}{F_{s}})(iR+N_{0})},$$

Substituting Equation (9) into Equation (6), the following equation is described as:

(10) $$\begin{array}{cc} S_{x}(\alpha ,f_{k};p)= & \frac{1}{K{\left\|w\right\|}^{2}F_{s}}\sum_{i=0}^{K-1}X_{w}(i,f_{k})X_{w}{\left(i,f_{k-p}\right)}^{*}e^{-j2\pi (\frac{\alpha }{F_{s}}-\frac{p}{N_{w}})(iR+N_{0})}\\ & =\frac{1}{K{\left\|w\right\|}^{2}F_{s}}\underset{i\to \alpha }{DFT}\left\{X_{STFT}(i,f_{k})X_{STFT}{\left(i,f_{k-p}\right)}^{*}\right\}e^{-j2\pi N_{0}(\frac{\alpha }{F_{s}}-\frac{p}{N_{w}})} \end{array}$$

If *p* = 0, the band [*f_k_*-Δ*f*/2, *f_k_*+Δ*f*/2] is flowed by the energy. Otherwise, the energy will flow between bands [*f_k_*-Δ*f*/2, *f_k_*+Δ*f*/2] and [*f_k_*_-*p*_-Δ*f*/2, *f_k_*_-*p*_+Δ*f*/2].

Therefore, the expression of fast spectral correlation is given by [24]:

(11) $$S_{x}^{Fast}(\alpha ,f)=\frac{\sum_{p=0}^{p}S_{x}(\alpha ,f;p)}{\sum_{p=0}^{p}R_{w}(\alpha -p\Delta f)}R_{w}(0),$$

where $R_{w}(\alpha )={\sum_{n=0}^{N_{w}-1}\left|w\left[n\right]\right|}^{2}e^{-j2\pi (n-N_{0})\frac{\alpha }{F_{s}}}$ represents the kernel function, and when *α=0* the expression of kernel function is as $R_{w}(0)={\left\|w\right\|}^{2}$. 

#### 2.2.2. Integrated Fast Spectral Correlation

In order to effectively separate the fault features, the integrated fast spectral correlation (IFSC) theory was proposed in this paper. The spectral correlation matrix **S** obtained by fast spectral correlation is an I×J matrix, whose dimension is expressed as frequencies × cyclic frequencies. After the spectral correlation matrix is obtained, the fourth-order energy at all the cyclic frequencies is added together, and the resonance band can be identified by observing the distribution of the accumulated energy along the frequency axis. In general, different resonance bands in complex faults represent different characteristics of faults. By observing the resonance band, the frequency range [*f*_1_, *f*_2_] of the resonance band is determined, and the spectral correlation matrix is integrated to obtain the integrated fast spectral correlation results. The expression of fourth-order energy is given by:

(12) $$E\left(i\right)=\sum_{j=1}^{J}S_{{}_{\left(i,j\right)}}^{4},$$

## 3. The Proposed Method 

Since the actual collected rolling bearing signal is usually covered by many noise components which have an interference effect on the extraction of fault features. In order to effectively improve signal-to-noise ratio and separate composite faults, the following steps are taken:

Step 1: Collect the composite fault signal.

Step 2: Singular negentropy difference spectrum is applied to separate the trend component which contains the fault feature information from the interference component.

Step 3: Fast spectral correlation is utilized to obtain the fast spectral correlation spectrum, the fourth-order energy of whose frequency axis is added together. As the result, the resonance band can be identified.

Step 4: Fast spectral correlation is used to separate different faults and obtain the enhanced envelope spectrum of each fault frequency band.

**Figure 3:**
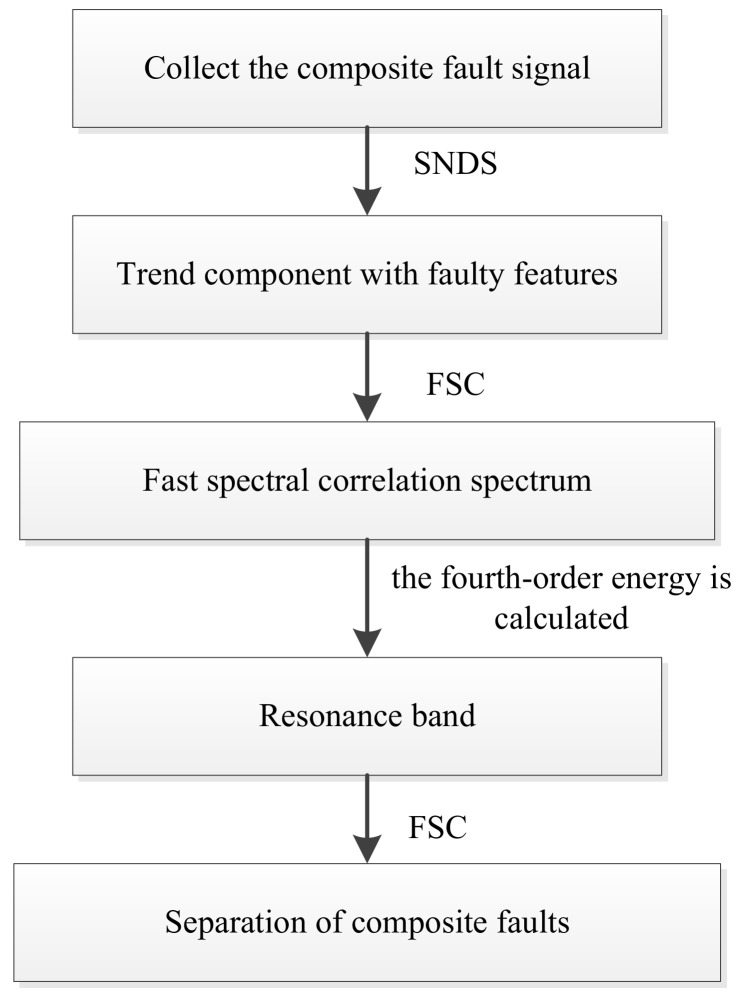
The flow chart of the proposed method.

The flow chart of the method presented in this paper is shown in Figure 3.

## 4. Simulation Analysis

In order to verify the effectiveness of the proposed method, a simulated composite fault signal of inner and outer ring is performed.

(13) $$\left\{\begin{array}{l} x_{1}(t)=3e^{-350t_{1}}\cdot \sin(2\pi f_{1}t),t_{1}=\mathrm{mod}(t,1/f_{i})\\ x_{2}(t)=2.5e^{-400t_{2}}\cdot \sin(2\pi f_{2}t),t_{2}=mod(t,1/f_{o})\\ x_{3}(t)=x_{1}(t)+x_{2}(t)\\ x(t)=x_{3}(t)+n(t) \end{array}\right..$$

where *x*_1_(*t*) is the simulation signal of rolling bearing with inner ring fault whose inner ring fault characteristic frequency is expressed as *f*_i_ = 130 Hz and natural frequency *f*_1_ is expressed as *f*_1_ = 3000 Hz. *x*_2_(*t*) is the simulation signal of rolling bearing with outer ring fault whose outer ring fault characteristic frequency is expressed as *f*_o_ = 90 Hz and natural frequency *f*_2_ is expressed as *f*_2_ = 1000 Hz. *n*(*t*) is white noise. In the simulation, the sampling frequency of the example signal is *f*_s_ = 8192 Hz and the sampling number is *N* = 4096.

**Figure 4:**
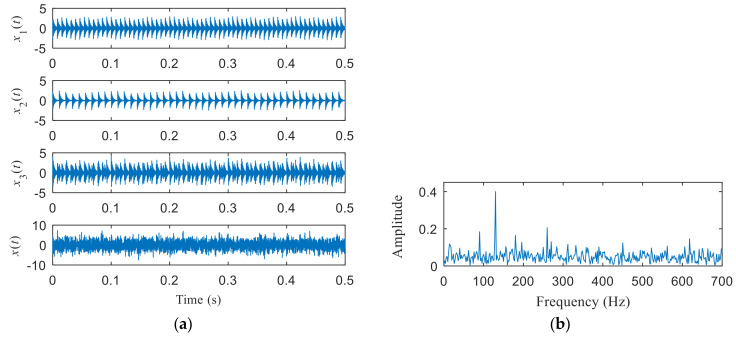
(**a**) Time domain waveform of the simulation signals; (**b**) the envelope spectrum of *x*(*t*).

**Figure 5:**
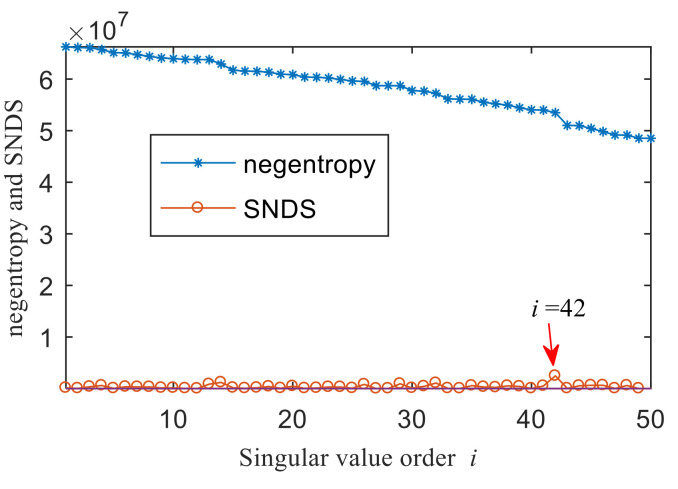
Negentropy of first 50 points and the singular negentropy difference spectrum (SNDS).

**Figure 6:**
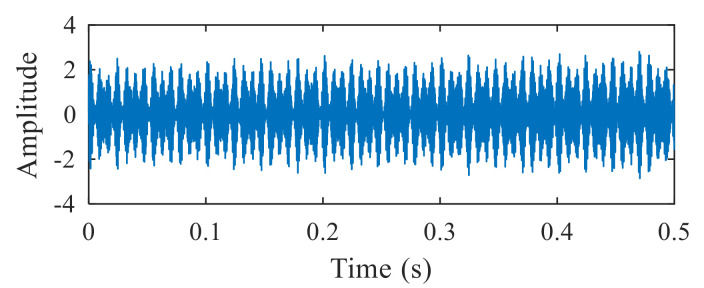
Time domain waveform of the reconstructed signal.

Figure 4a shows the time domain waveform of the simulation signal. It can be seen from Figure 4a that the simulation signals *x*_1_(*t*), *x*_2_(*t*) and *x*_3_(*t*) all contain periodic impulse components, while the periodic impulse components in *x*(*t*) have been submerged by noise. Figure 4b shows that the envelope spectrum of *x*(*t*) has no prominent frequency components. The Hankel matrix is constructed from the original signal and processed by SVD. The negentropy of first 50 points and singular negentropy difference spectrum are shown in Figure 5. It can be found that the 42th point is the maximum mutation points of the difference spectrum, retaining the first 42 singular values obtained by SVD processing, and the other singular values are all set to 0. The reconstruction signal shown in Figure 6 is obtained by singular value reconstruction. Compared with Figure 6 and Figure 4a, the periodic impulse components are more obvious in the time domain waveform after SVD reconstruction.

**Figure 7:**
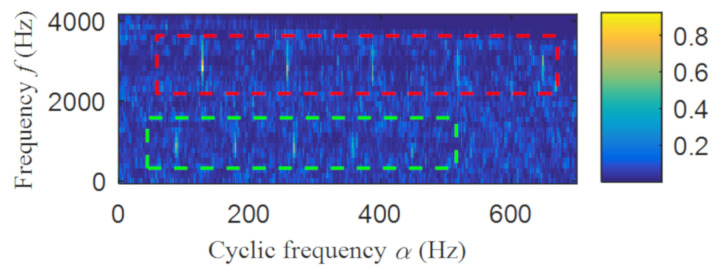
The fast spectral correlation spectrum of the reconstructed signal.

**Figure 8:**
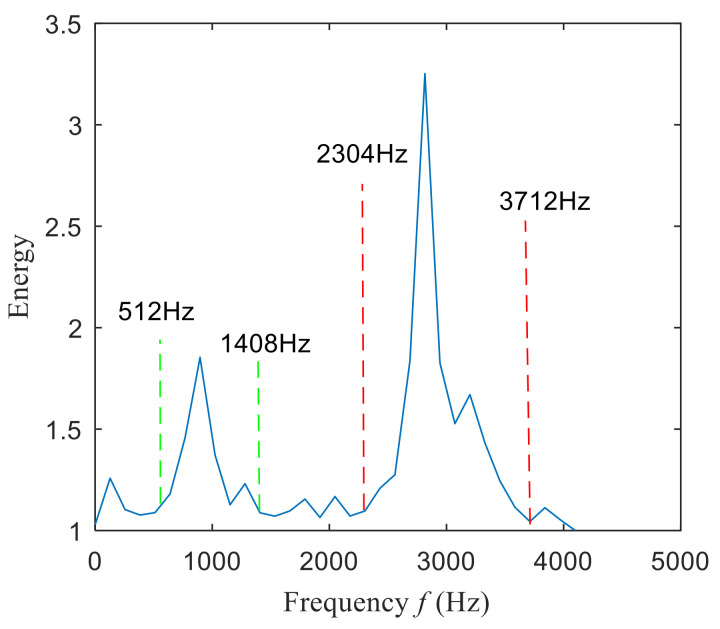
Screening of resonance bands

**Figure 9:**
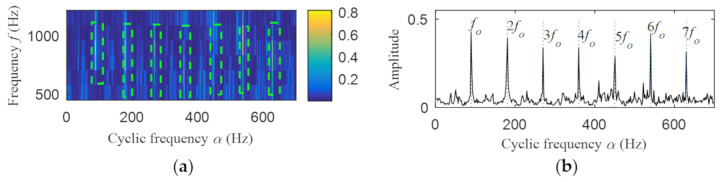
Outer ring fault after separation: (**a**) the fast spectral correlation spectrum; (**b**) the enhanced envelope spectrum.

**Figure 10:**
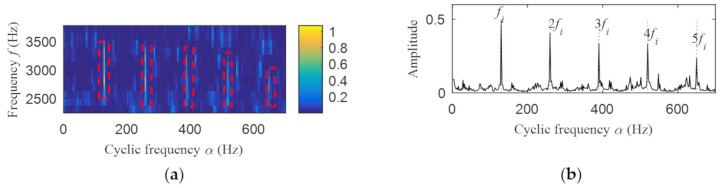
Inner ring fault after separation: (**a**) the fast spectral correlation spectrum; (**b**) the enhanced envelope spectrum.

Then, the fast spectral correlation is performed on the reconstructed signal, and the fast spectral correlation spectrum shown in Figure 7 is obtained. Two resonance bands are found in Figure 7, among which the red box area means the resonance band of the inner ring fault and the green box area is the resonance band of the outer ring fault. Next, the fourth-order energy of the ordinate in Figure 7 is carried out to obtain the resonance band screening diagram shown in Figure 8. According to Figure 8, the two resonance bands are 512–1408 Hz and 2304–3712 Hz, respectively. Fast spectral correlation is conducted for the frequency ranges of the two resonance bands respectively, the outer and inner ring faults after separation are finally obtained as shown in Figure 9 and Figure 10. Figure 9 describes the fast spectral correlation and enhanced envelope spectrum of outer ring fault, from which *f*_o_, 2*f*_o_, 3*f*_o_, 4*f*_o_, 5*f*_o_, 6*f*_o_ and 7*f*_o_ can be recognized obviously. Figure 10 describes the fast spectral correlation and enhanced envelope spectrum of inner ring fault. *f*_i_ and its 2X, 3X, 4X, 5X frequency doubling can be clearly identified from Figure 10.

**Figure 11:**
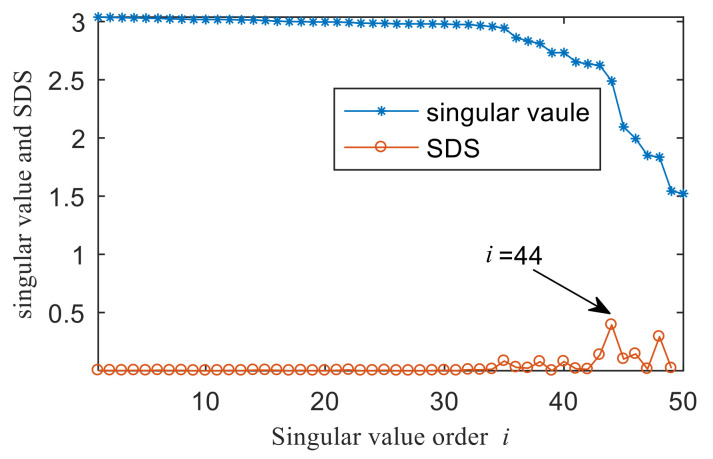
Singular value of first 50 points and singular difference spectrum (SDS).

**Figure 12:**
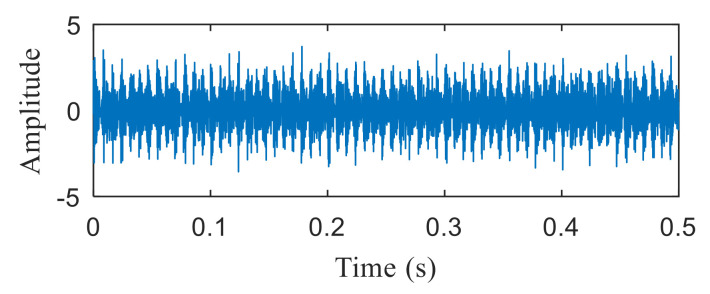
Time domain waveform of the reconstructed signal.

**Figure 13:**
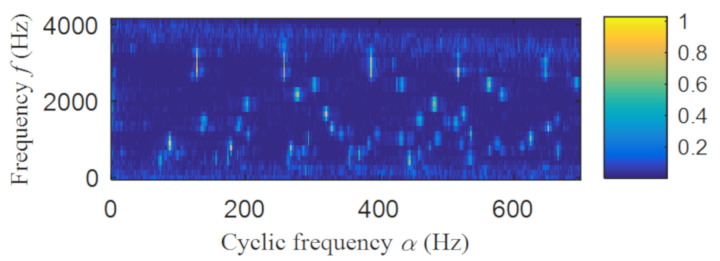
The fast spectral correlation spectrum of the reconstructed signal.

In order to show the superiority of the proposed method, the integrated fast spectral correlation based on singular difference spectrum and spectral kurtosis based on wavelet [26] are used to analyze the simulation signal. Figure 11 shows the singular difference spectrum of the simulation signal. As shown in Figure 11, *i* = 44 is the mutation point. The singular values after *i*= 44 are set to 0, and the time domain diagram of the reconstructed signal is shown in Figure 12. While the periodic impulse in the reconstructed signal is more obvious than that in the simulation signal, it still contains some noise components. The fast spectral correlation spectrum is shown in Figure 13. Compared with Figure 7, the resonance bands of outer and inner ring faults cannot be clearly identified.

**Figure 14:**
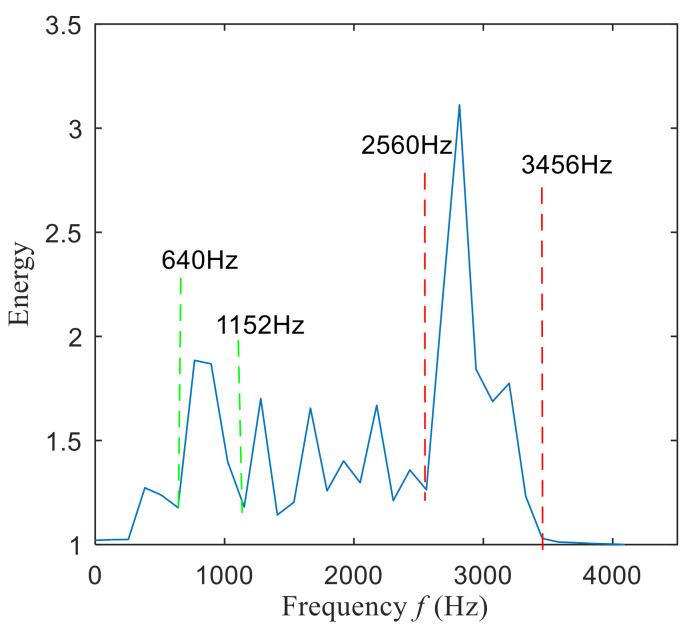
Screening of resonance bands

**Figure 15:**
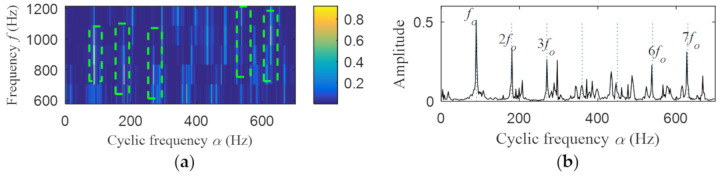
Outer ring fault after separation: (**a**) the fast spectral correlation spectrum; (**b**) the enhanced envelope spectrum.

**Figure 16:**
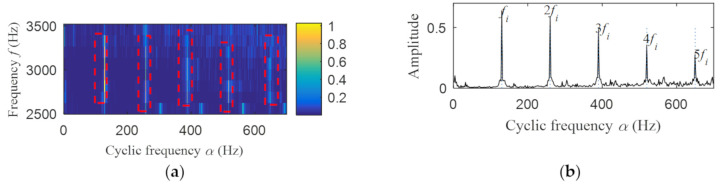
Inner ring fault after separation: (**a**) the fast spectral correlation spectrum; (**b**) the enhanced envelope spectrum.

Figure 14 shows the separation of resonance bands, from which, the inner fault resonance band can be accurately identified, while the outer fault resonance band cannot be clearly identified. Figure 15 shows the fast spectral correlation and enhanced envelope spectrum of outer ring fault. From Figure 15a,b, only *f*_o_, 2*f*_o_, 3*f*_o_, 6*f*_o_ and 7*f*_o_ can be recognized. It can be also found from Figure 15b that there are many interference components. The fast spectral correlation and enhanced envelope spectrum of inner ring fault is shown in Figure 16. *f*_i_ and its 2X, 3X, 4X, 5X frequency doubling can be clearly identified from Figure 16. Compared with Figure 9 and Figure 10, the separation effect of the proposed method is better than that of the integrated fast spectral correlation based on singular difference spectrum.

**Figure 17:**
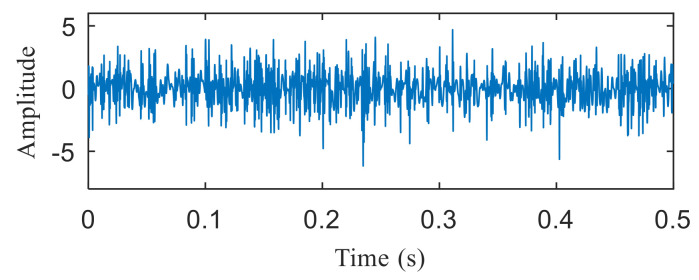
Time-domain waveform after wavelet denoising.

**Figure 18:**
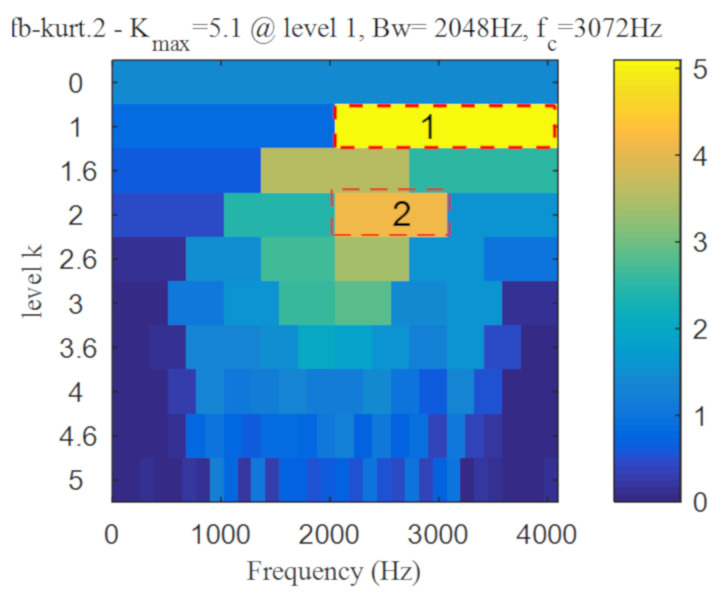
Kurtogram of the denoised signal.

**Figure 19:**
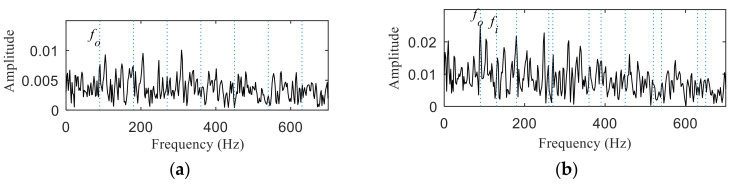
The results of separation: (**a**) the envelope spectrum of band 1; (**b**) the envelope spectrum of band 2.

As shown in Figure 17, periodic impact components cannot be noticed in the time-domain waveform after wavelet denoising. Kurtogram of the denoised signal is shown in Figure 18, and two frequency bands was selected according to it. It can be seen from Figure 19a,b, the envelope spectrums contain the characteristic frequency of inner ring and outer ring, but the composite fault is not separated into single ones. The results show that the proposed method has a better separation performance than the method combing wavelet transform and spectral kurtosis.

## 5. Experimental Analysis

### 5.1. Introduction of Experiment

**Figure 20:**
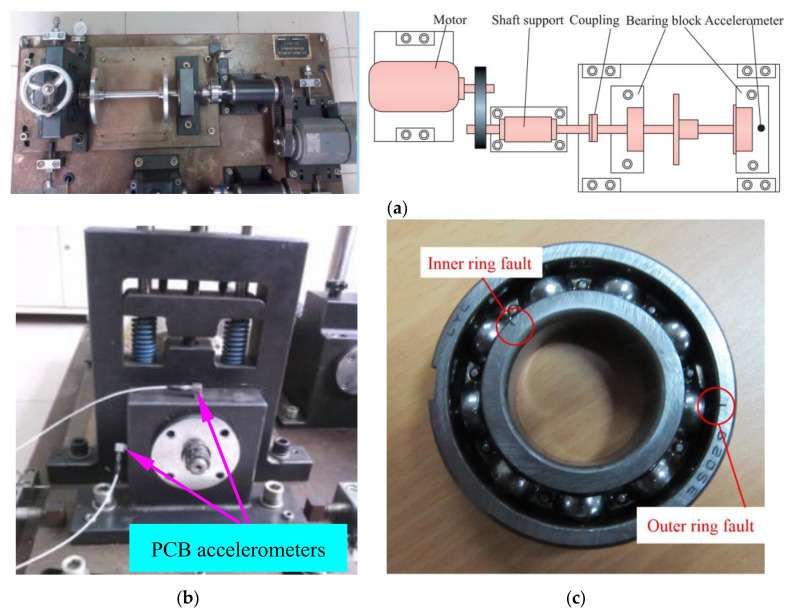
(**a**) Experiment stand; (**b**) sensors distribution and (**c**) rolling bearing with inner ring fault and outer ring fault.

In order to prove the effectiveness of the proposed method in practical application, QPZZ model test-bed is applied to simulate the composite fault of inner and outer rings of rolling bearings. Figure 20a, b and c show the experimental devices, sensor distribution and composite faults of inner and outer rings, respectively. The test bench is mainly composed of a motor, a shaft support, coupling, bearings, block and accelerometers. The geometric parameters of rolling bearings are shown in Table 1. The speed of motor is 1470 r/min during the experiment. The sampling frequency *f_s_* is 12,800 Hz, the sampling point *N* is 6400 and the rotating frequency *f_r_* is 24.5 Hz. The characteristic frequency of inner ring fault *f*_i_ is 132 Hz and the outer ring fault characteristic frequency *f*_o_ is 88 Hz.

### 5.2. Compound Fault Diagnosis

**Figure 21:**
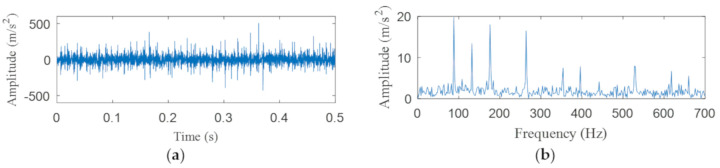
(**a**) Time domain waveform of the original signal; (**b**) the envelope spectrum of the original signal.

**Figure 22:**
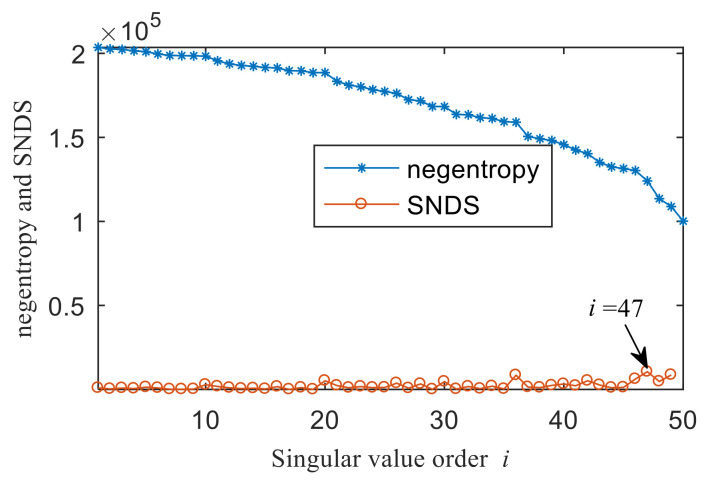
Negentropy of the first 50 points and singular negentropy difference spectrum (SNDS).

**Figure 23:**
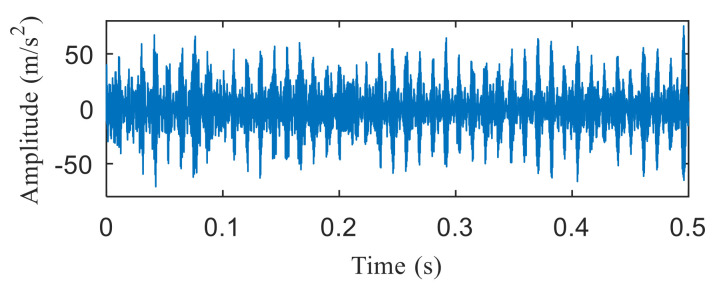
Time domain waveform of the reconstructed signal.

Figure 21a,b are the time domain waveform and envelope spectrogram of the original signal, respectively. From the time-domain waveform of the original signal, we can see that it contains many noise components, which cannot be identified from the envelope spectrum. What is more, the fault type cannot be determined from Figure 21b. Figure 22 shows the negentropy and singular negentropy difference spectrum of the signal reconstructed from the first i-th singular values after SVD decomposition. From Figure 22, we can see that i = 47 is the mutation position of negentropy value. Hence, the negentropy values after i= 47 are set to 0. The signal is reconstructed, then the time domain waveform of Figure 23 is obtained. 

**Figure 24:**
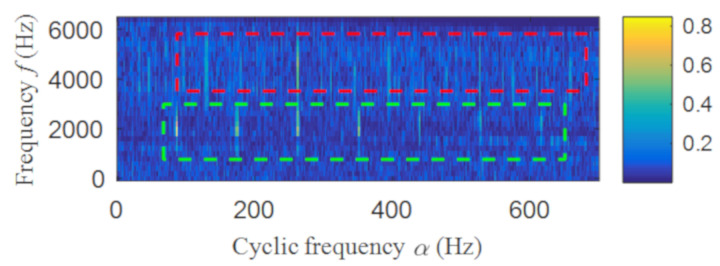
The fast spectral correlation spectrum of the reconstructed signal.

**Figure 25:**
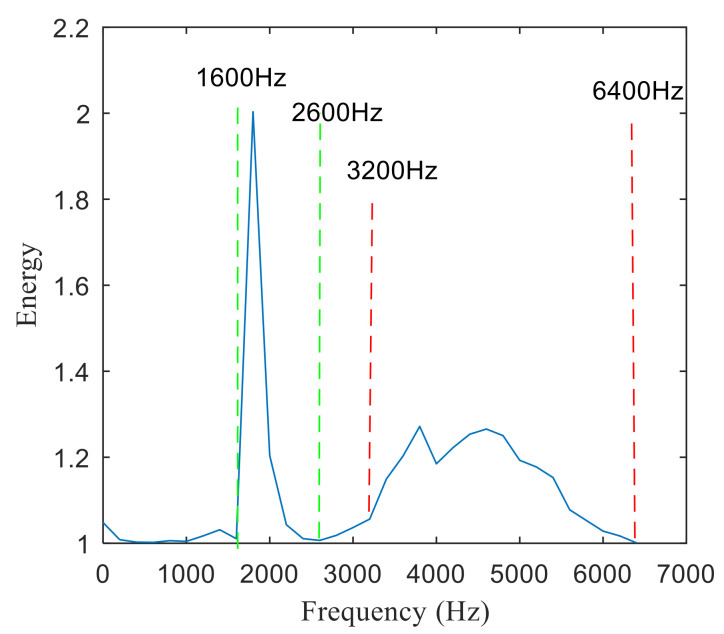
Screening of resonance bands.

**Figure 26:**
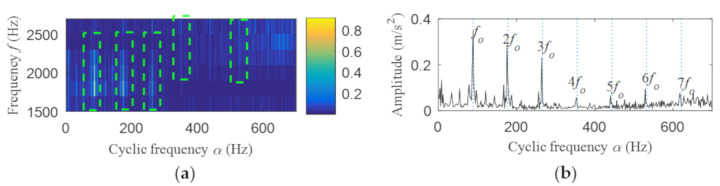
Outer ring fault after separation: (**a**) the fast spectral correlation spectrum; (**b**) the enhanced envelope spectrum.

**Figure 27:**
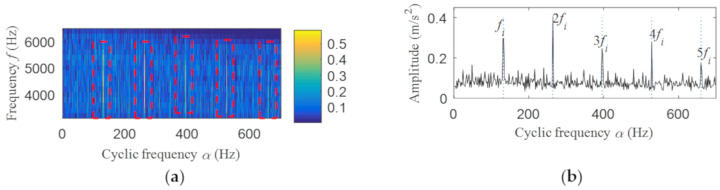
Inner ring fault after separation: (**a**) the fast spectral correlation spectrum; (**b**) the enhanced envelope spectrum.

The fast spectral correlation analysis of the reconstructed signal is carried out, and the fast spectral correlation spectrum of Figure 24 is obtained. From Figure 24, two resonance bands are found, among which the green rectangular frame denotes the resonance band of the outer ring fault and the red rectangular frame means the resonance band of the inner ring fault. By calculating the fourth-order energy of the frequency of Figure 24, the resonance bands of the outer and inner ring faults can be separated. It can be seen from Figure 25 that the resonance band of outer ring fault is 1600 Hz–2600 Hz and the inner ring fault is 3200 Hz–6400 Hz. Fast spectral correlation analysis of the two resonance bands is carried out, and then the fast spectral correlation results shown in Figure 26 and Figure 27 are obtained.

**Figure 28:**
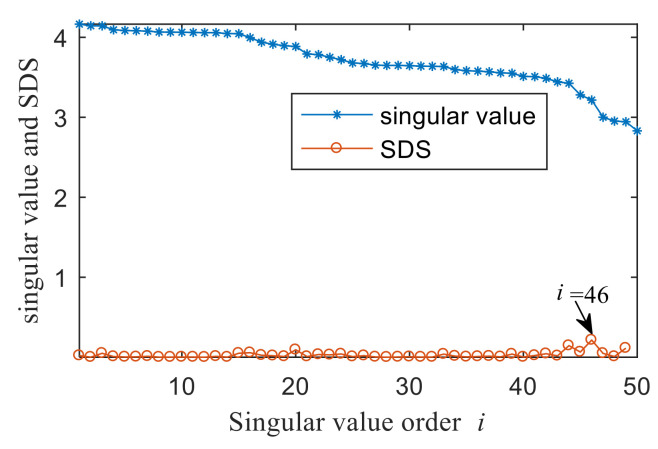
Singular value of the first 50 points and singular difference spectrum (SDS).

**Figure 29:**
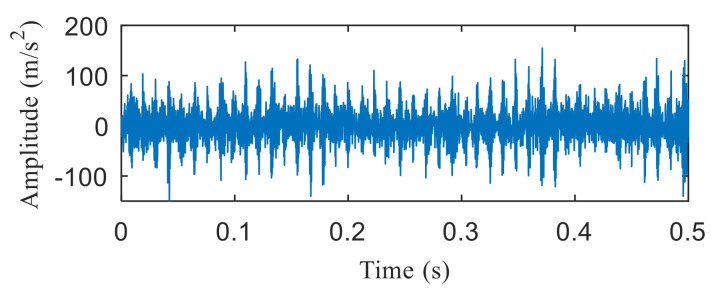
Time domain waveform of the reconstructed signal.

**Figure 30:**
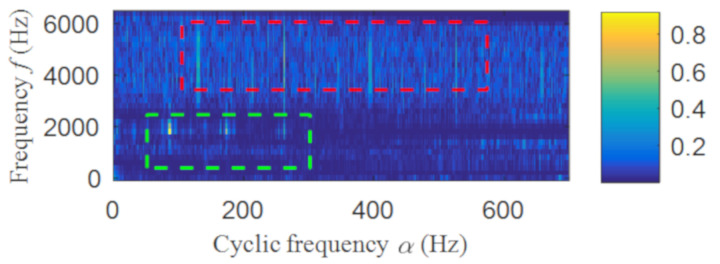
The fast spectral correlation spectrum of the reconstructed signal.

**Figure 31:**
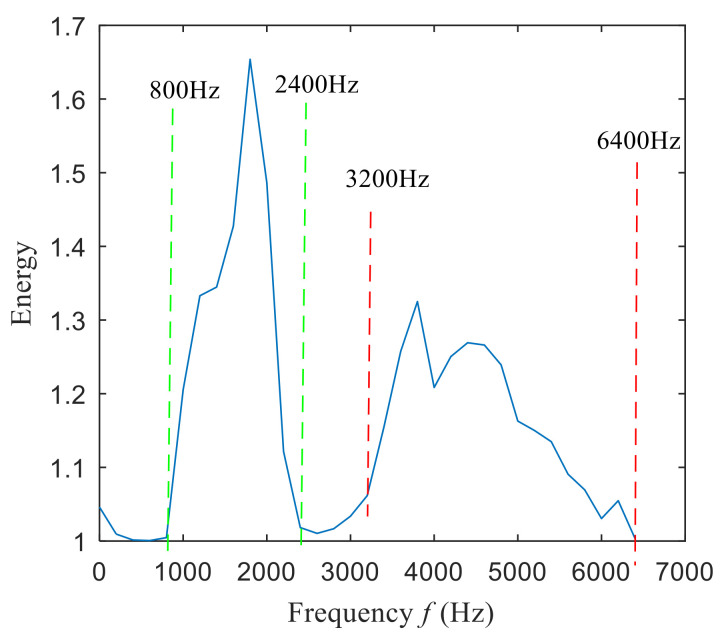
Screening of resonance bands.

**Figure 32:**
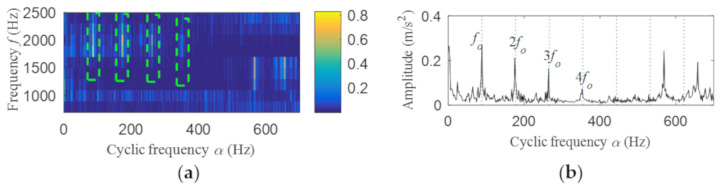
Outer ring fault after separation: (**a**) the fast spectral correlation spectrum; (**b**) the enhanced envelope spectrum.

**Figure 33:**
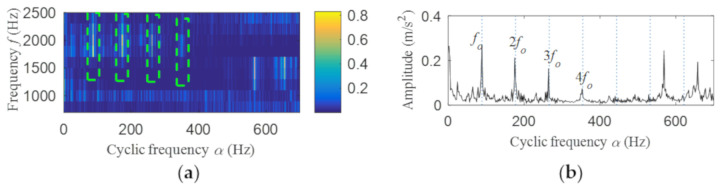
Inner ring fault after separation: (**a**) the fast spectral correlation spectrum; (**b**) the enhanced envelope spectrum.

The result of integrated fast spectral correlation method based on singular difference spectrum is compared to show the superiority of SNDS. Figure 28 shows the singular difference spectrum of the reconstructed signals which is composed of the first *i*-th singular values. As shown in Figure 28, *i*=44 is the mutation point. The singular values after *i* = 44 are set to 0, and the time domain diagram of reconstructed signal is shown in Figure 29. Although the periodic pulse in reconstructed signal is more obvious than that in simulated signal, it still contains some noise components. The fast spectral correlation spectrum is shown in Figure 30. Compared with Figure 24, the resonance bands of outer and inner ring faults cannot be clearly identified. Figure 31 shows the separation of the resonance band. It can be seen from Figure 31 that the internal fault resonance band can be accurately identified, while the external fault resonance band cannot be clearly identified. Figure 32 shows the fast spectral correlation and enhanced envelope spectrum of the outer ring fault. From Figure 32a,b, we can only identify *f*_o_, 2*f*_o_, 3*f*_o_ and 4*f*_o_. Figure 33 describes the fast spectral correlation and enhanced envelope spectra of inner ring faults. *f*_i_ and its 2X, 3X, 4X and 5X multiples can be clearly identified in Figure 33. Compared with Figure 26 and Figure 27, the separation effect of this method is better than that of the integrated fast spectral correlation method based on singular difference spectrum.

**Figure 34:**
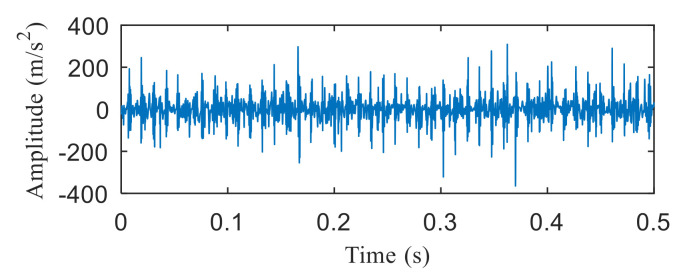
Time-domain waveform after wavelet denoising.

**Figure 35:**
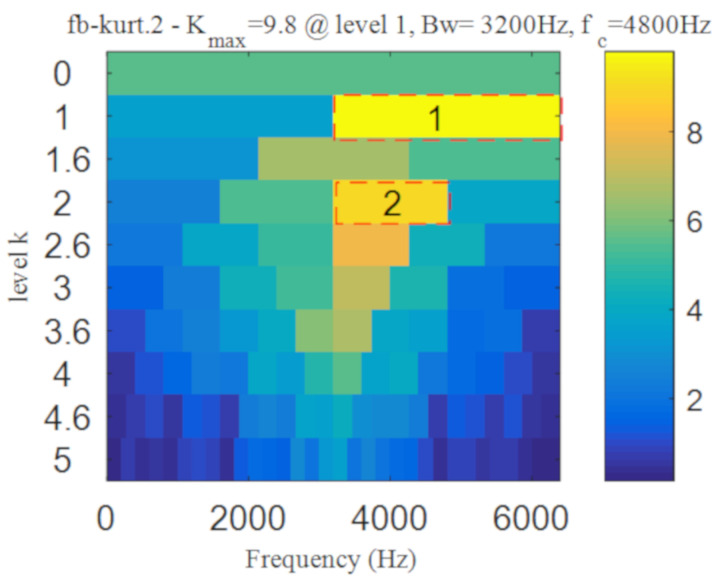
Kurtogram of denoised signal.

**Figure 36:**
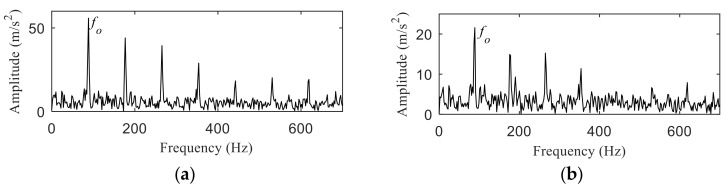
The results of separation: (**a**) the envelope spectrum of band 1; (**b**) the envelope spectrum of band 2.

Similar to simulation analysis, the method based on wavelet transform and spectral kurtosis was performed to compare with the proposed method. It can be seen from Figure 34 that the period impact components are more obvious after wavelet denoising. The two frequency bands with the strongest impact were selected from Kurtogram shown in Figure 35. The fault with weak impact is easy to be submerged by the fault with strong impact. Hence, as shown in Figure 36, only the outer ring fault is separated. The frequency of inner ring fault cannot be identified from both Figure 36a,b. Comparing Figure 26 and Figure 27 with Figure 36, the proposed method has superior capability to separate the composite faults than the method based on wavelet transform and spectral kurtosis.

## 6. Conclusions

In this paper, a compound fault feature separation method based on singular negentropy difference spectrum and integrated fast spectral correlation was proposed. Singular negentropy difference spectrum was applied to de-noise and then integrated fast spectral correlation was performed. The results show that the composite fault signal can be separated effectively and accurately. The following conclusions can be drawn:

(1) Singular negentropy difference spectrum (SNDS) can adaptively determine the effective singular value, effectively remove the noise components and retain useful fault information. What is more, the comparison between SNDS and the singular difference spectrum shows that SNDS has better denoising performance.
(2) The fourth-order energy was used as the index by integrated fast spectrum correlation (IFSC) to select different resonance bands, so as to realize the separation of different faults. The method combining wavelet transform and spectral kurtosis was used to compare with the proposed method in this paper, the results show that the proposed method can separate the composite faults better.
(3) Limited to the experimental conditions, the composite fault diagnosis of rolling bearing is only discussed in this paper. There are many other composite faults of rotating machines, such as compound fault of gear, compound fault of gear and rolling bearing. In the future, we will continue to study the difficult problem of fault diagnosis of other fault modes.

## Figures and Tables

**Table 1:** Specific information about the experiment bearings.

Type	Diameter of Balls, *d* (mm)	Pith Diameter, *D* (mm)	Number of Balls, *z*	Contact Angle, *α* (°)	Damage Size of Inner Ring, (mm)	Damage Size of Outer Ring, (mm)
SKF6205	7.5	38.5	9	0	0.008	0.059
